# Clinical and Economic Impact of a Digital, Remotely-Delivered Intensive Behavioral Counseling Program on Medicare Beneficiaries at Risk for Diabetes and Cardiovascular Disease

**DOI:** 10.1371/journal.pone.0163627

**Published:** 2016-10-05

**Authors:** Fang Chen, Wenqing Su, Shawn H. Becker, Mike Payne, Cynthia M. Castro Sweet, Anne L. Peters, Timothy M. Dall

**Affiliations:** 1 IHS Life Sciences, Washington, District of Columbia, United States of America; 2 Silvercat Advisors, LLC, Burlingame, California, United States of America; 3 Omada Health, Inc., San Francisco, California, United States of America; 4 Keck School of Medicine of University of Southern California, Los Angeles, California, United States of America; Florida International University Herbert Wertheim College of Medicine, UNITED STATES

## Abstract

**Background:**

Type 2 diabetes and cardiovascular disease impose substantial clinical and economic burdens for seniors (age 65 and above) and the Medicare program. Intensive Behavioral Counseling (IBC) interventions like the National Diabetes Prevention Program (NDPP), have demonstrated effectiveness in reducing excess body weight and lowering or delaying morbidity onset. This paper estimated the potential health implications and medical savings of a digital version of IBC modeled after the NDPP.

**Methods and Findings:**

Participants in this digital IBC intervention, the Omada program, include 1,121 overweight or obese seniors with additional risk factors for diabetes or heart disease. Weight changes were objectively measured via participant use of a networked weight scale. Participants averaged 6.8% reduction in body weight within 26 weeks, and 89% of participants completed 9 or more of the 16 core phase lessons. We used a Markov-based microsimulation model to simulate the impact of weight loss on future health states and medical expenditures over 10 years. Cumulative per capita medical expenditure savings over 3, 5 and 10 years ranged from $1,720 to 1,770 (3 years), $3,840 to $4,240 (5 years) and $11,550 to $14,200 (10 years). The range reflects assumptions of weight re-gain similar to that seen in the DPP clinical trial (lower bound) or minimal weight re-gain aligned with age-adjusted national averages (upper bound). The estimated net economic benefit after IBC costs is $10,250 to $12,840 cumulative over 10 years. Simulation outcomes suggest reduced incidence of diabetes by 27–41% for participants with prediabetes, and stroke by approximately 15% over 5 years.

**Conclusions:**

A digital, remotely-delivered IBC program can help seniors at risk for diabetes and cardiovascular disease achieve significant weight loss, reduces risk for diabetes and cardiovascular disease, and achieve meaningful medical cost savings. These findings affirm recommendations for IBC coverage by the U.S. Preventive Services Task Force.

## Introduction

Intensive Behavioral Counseling (IBC) has been widely accepted as an effective approach for compliant individuals to reduce body weight, improve clinical measures, and lower risk of obesity-related comorbidities [1–6]. The US Preventive Services Task Force (USPSTF) recommends that, as part of cardiovascular diseases (CVD) risk assessment, all overweight and obese adults between age 40 and 70 be screened for abnormal blood glucose, and those with CVD risk factors (e.g., hypertension, dyslipidemia, prediabetes) be referred for IBC, such as programs modeled on the National Diabetes Prevention Program (NDPP) [3,5].

Outcomes from the Diabetes Prevention Program and Outcomes Study (DPPOS) have shown that at 3 years of follow up, lifestyle intervention successfully reduced diabetes incidence by 58% and reduced CVD risk with significantly less use of hypertension and hyperlipidemia medication [3]. Furthermore, the 10- and 15-year follow-up studies continued to show reduced incidence of diabetes by 34% and 27%, respectively, relative to the control group [1,2]. Although these studies did not directly report outcomes on long-term CVD events, epidemiological and pathological data have shown that obesity and diabetes are risk factors for cardiovascular disease [7,8].

A systematic literature review sponsored by the Agency for Healthcare Research and Quality concluded that comprehensive lifestyle interventions extend benefits beyond the intervention phases including improved metabolic levels and reduced incidence of type 2 diabetes [6]. The study concluded that secondary outcomes (improved metabolic measures) could be generalized to the senior population (age ≥ 65 years), with evidence that weight loss intervention for older adults is effective in improving health outcomes [9,10].

The economic costs associated with cardiovascular disease and diabetes are significant, and without intervention are expected to increase because of the aging US population. In 2012, the direct medical costs of diabetes were estimated to be $176 billion. Coronary heart disease-related health care costs are projected to rise by 41% from $126 billion in 2010 to $176 billion in 2040. These statistics emphasize the need to find ways to reduce the burden of disease in both CVD and diabetes through prevention and treatment [11,12].

A digital, remotely-delivered IBC program that is an enhanced version of the original NDPP demonstrated clinical efficacy [13–16]. Among the general adult population with either prediabetes or CVD risk factors, this IBC reduced average body weight approximately 4–6% (depending on level of program participation and demographics of the participants) and reduced average hemoglobin A1c levels approximately 0.4% [13,15]. Among a general adult population, estimates of medical savings per person exceeded $1,300 within three years, and benefits continued growing to $8,000-$9,000 cumulative over 10 years [14].

The goal for this paper was to model the impact of a digital IBC for senior participants (ages 65 or over) in order to estimate the potential clinical and economic benefits for the Medicare program.

## Methods

We provide a brief description of the intervention and population analyzed, and an overview of the simulation model used to estimate the clinical and economic implications of participation. The Western Institutional Review Board’s IRB Affairs Department confirmed the study met the conditions for exemption and consent procedure was not recorded.

### Intervention and Study Sample

This study analyzed senior participants in a digital IBC intervention, the Omada program (Omada Health, San Francisco, California). Features of the program include: an initial intensive 16-week online educational program followed by an ongoing program designed to sustain the behavioral changes; social network support in which participants are demographically matched into online communities to promote accountability and learning via shared experience; a professional health coach to facilitate group interactions and provide individual counseling; digital tracking tools that include a wireless scale from which data is automatically uploaded into the patient’s record and a pedometer; and 24 x7 telephone and online availability. The program exceeds the Centers for Disease Control and Prevention (CDC) Diabetes Prevention Recognition Program standards and eliminates the need for patients to travel to a “brick and mortar” program [14,17].

The study sample consisted of 1,121 adults age 65 and over who enrolled between 2012 and 2015, for at least 16 weeks, and had baseline body mass index (BMI) of 24 kg/m2 or higher [17]. These enrollees constitute the intent-to-treat (ITT) cohort. We also analyzed program completers (participants who completed ≥ 9 lessons, N = 1,003) using CDC guidelines to define a completer [17]. Each participant’s weight change was calculated as the difference between beginning body weight and the last recorded weight between weeks 17 and 26. Information recorded for each individual consisted of age, sex, race and ethnicity, beginning weight, weight change, and whether the individual had prediabetes. All individuals met the USPSTF criteria for receiving IBC because they had CVD risk factors independent of excess body weight; with 997 (89%) also meeting the CDC and NDPP recommendations for intervention due to prediabetes [3,5,17].

### Simulation Model

Our simulation model (described later) requires information beyond the data collected by the IBC program described above—including hemoglobin A1c level, systolic blood pressure (SBP) and diastolic blood pressure (DBP), total cholesterol and high-density lipoprotein cholesterol (HDL-C), current smoking status, presence of medical conditions linked to obesity, and use of medications to treat hypertension and hypercholesterolemia. To fill in missing data required to run the simulation, we used a 1:1 propensity score to match each program participant to a similar person in the combined 2005–2012 National Health and Nutrition Examination Survey (NHANES) files based on age, sex, race and ethnicity, starting BMI, prediabetes status, and presence of CVD risk factors.

When using the 1:1 propensity matching we matched each digital IBC participant to NHANES samples in one of two populations:

1. *Prediabetes population*. 997 out of 1,121 studied participants with prediabetes were matched to individuals from NHANES with prediabetes (A1c 5.7%–6.4%) that have similar demographics and BMI.
2. *CVD risk population*. This is the entire participant sample that combines the above prediabetes population and remaining 124 participants without prediabetes. The remaining participants were matched to NHANES individuals with similar demographics and BMI, who did not have prediabetes or diabetes, but who had at least one other CVD risk factor available in NHANES (hypertension, hyperlipidemia, and tobacco use).

For each of these populations we analyzed all enrollees, as well as the subset that completed at least 9 lessons in the program.

### Simulation modeling

**Fig 1:**
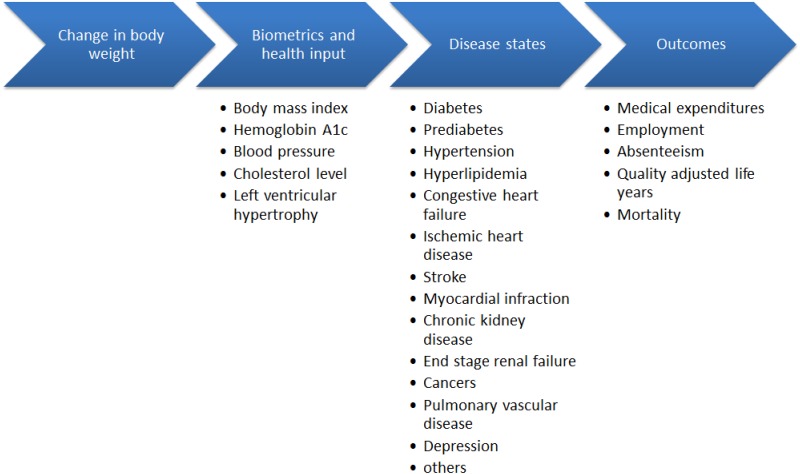
Model Overview Diagram.

We used a Markov-based microsimulation approach to quantify the impact of weight change on individuals’ health states and medical expenditures [14,18–20]. The model uses an annual cycle, with each person’s current health status used to predict the upcoming year’s outcomes (Fig 1). We ran the simulation under three scenarios:

*Intervention scenario 1 (sustained weight loss)*: Each person’s weight change realized through the digital IBC program occurred at the beginning of the first simulation year. From years 2 through 10 year-to-year weight change followed trends in the U.S. adult population for people of similar age and weight (i.e., natural history of weight change). Simulation results suggest this senior population will continue to lose 0.2% of their initial body weight each year.

*Intervention scenario 2 (partial weight regain)*: Each person’s weight change realized through the digital IBC program occurred at the beginning of the first simulation year. However, during years 2 through 6 the person regained about one third of the weight loss (similar to patterns observed in years 2 through 6 of NDPP) and maintained approximately two thirds of the original weight loss through year 10. Published 2-year results of this digital IBC program suggest weight regain was less aggressive than the original NDPP study [13,17].

*Control scenario*: No weight loss occurs, and each person’s year-to-year weight change follows the natural history of weight change. Differences between the control scenario and intervention scenario 1 (sustained weight loss) outcomes reflect optimistic estimates of program impact, while differences between control scenario and intervention scenario 2 (partial weight regain) represent conservative estimates of program impact. The maintenance phase of the program is designed to help patients sustain the outcomes achieved under the core phase. Hence, the overall intervention impact is likely bounded by intervention scenarios 1 and 2. (Fig 1)

In the simulation model, change in body weight affects A1c, blood pressure, and cholesterol levels (as well as having an independent effect on disease states described below). In turn, these clinical outcomes—combined with demographics, BMI, smoking status, and presence of chronic conditions—are used in prediction equations for various disease onset, severity, and mortality. Equations predicting annual alteration in biometric parameters and disease states are based on published clinical and observational studies. For example, the correlation between mean change in body weight and mean change in A1c levels comes from the CONQUER trial—a randomized clinical trial that enrolled individuals with BMI of 27–45 in the U.S. between 2007 and 2009 [21]. Over the 56 week period, each 1kg decrease in excess body weight was associated with a 0.071% decrease in A1c [22]. Meta-analysis of clinical trial outcomes suggests each 1kg loss in body weight reduced SBP by 1.05-mmHg [23]. The annual change in total cholesterol level is the combined effect from BMI change and aging, which were predicted separately using polynomial regression equations derived based on findings from Wilson et al [24]. Data sources for change in key biometric parameters are provided in S1 Table. Detailed technical documentation of the model prediction equations for disease onset, progression and mortality, data and assumptions that underlie the model, calculations for health economic outcomes, and validation results can be found in previous publications [18,19]. Sources for these prediction equations include studies such as Look AHEAD [19,25], UK Prospective Diabetes Study (UKPDS) [26–30], Framingham Heart Study [24,31,31–35], and other published clinical trials and observational studies [18,19].

### Direct medical cost

The equations simulating medical expenditures for each participant were estimated using the 2009–2013 Medical Expenditure Panel Survey (MEPS)—a nationally representative of the non-institutionalized population in the U.S. All medical costs were converted to 2015 dollars by using the medical component of the consumer price index.

We used a generalized linear model with gamma distribution and log link to model total annual medical expenditures for the population age 65 and older. The dependent variable was total annual medical expenditures. Explanatory variables include demographics (age group, sex, race, ethnicity); presence of diabetes, hypertension, congestive heart failure, ischemic heart disease, retinopathy, and end-stage renal disease; history of myocardial infarction, stroke, and various cancers; smoking status; and body weight. Separate regressions were estimated for the obese population (which included BMI as a continuous variable) and for the remaining population that was overweight or normal weight (S2 Table). In addition, mortality related expenditure represents the cost of medical care at the last year of life [36].

Following the approach of Faes et al. [37], we used a zero-inflated log-ratio regression to model the allocation of total medical expenditures across cost categories: ambulatory care, inpatient care, emergency care, prescription drug costs, and “all other” medical expenditures. These categories help illustrate the expected impact of lifestyle intervention on medical expenditures associated with Medicare Part A (inpatient), Part B (emergency and ambulatory) and Part D (prescription drug). An appendix summarizes technical detail and regression coefficients (S3 Table).

### Intervention cost

Gross medical cost saving is the accumulative saving from medical expenditure over years with a yearly discount rate of 3%. Net medical cost savings were calculated based on the average program cost for an active participant of the intervention program, which was estimated at $1,300 over 3 years. This assumes program costs of $400 upon program enrollment, $400 in year 1, $300 in year 2, and $200 in year 3.

## Results

### Digital IBC Outcomes

Of the senior participants in this study of a digital IBC program, 1,121 (100%) had risk factors for cardiovascular disease, and 997 (88.9%) had prediabetes (Table 1). Among the total sample, the average age was 69.0 years (standard error [SE], 0.13 years), 36% were male, and average starting BMI was 32.5 kg/m^2^ (SE, 0.16).

**Table 1:** Characteristics of Population with Prediabetes and Population at risk for Cardiovascular Disease at Baseline.

Patient Characteristics^a^	Population With Prediabetes		Population With CVD Risk Factors	
	Total (N = 997)	Completers^b^ (N = 896)	Total (N = 1,121)	Completers^b^ (N = 1,003)
Age, mean (SE), y	69.0 (0.12)	68.9 (0.12)	69.0 (0.13)	68.9 (0.11)
BMI, mean (SE), kg/m^2^	32.5 (0.17)	32.5 (0.17)	32.5 (0.16)	32.4 (0.16)
SBP, mean (SE), mm Hg	134.5 (0.65)	134.1 (0.70)	135.0 (0.65)	134.4 (0.70)
HDL-C, mean (SE), mg/dL	51.7 (0.40)	51.8 (0.45)	51.9 (0.40)	51.9 (0.42)
T-C, mean (SE), mg/dL	201.0 (1.49)	200.6 (1.56)	200.8 (1.39)	200.2 (1.45)
Hemoglobin A1c, mean (SE), %	6.0 (0.01)	6.0 (0.01)	6.0 (0.01)	6.0 (0.02)
Male, %	35.0	35.0	36.0	36.0
Prediabetes, %	100	100	88.9	89.3
Diabetes, %	0	0	0	0

Abbreviations: BMI, body mass index; HDL-C, high-density lipoprotein cholesterol; SBP, systolic blood pressure; SE, standard error; T-C, total cholesterol.

^a^ Values of SBP, HDL-C, T-C, Hemoglobin A1c and percentages of prediabetes/diabetes were estimated from a population with individuals matched from the National Health and Nutrition Examination Survey.

^b^ Participants completing ≥9 lessons.

Average weight loss recorded on or before week 26 was 6.8% (SE, 0.002). The large majority of participants (89.5%) successfully completed 9 or more core lessons, and characteristics of this subgroup were similar to total enrollees. The completers, however, achieved higher average weight loss of 7.3% (SE, 0.002).

### Model Simulation Results

In the Markov-based microsimulation model, the constructed population with prediabetes had an average A1c of 6.0% (SE, 0.01). Under the partial weight regain scenario (Scenario 2), simulated declines in diabetes onset among participants were 38.1%, 26.6%, and 11.1% over 3, 5, and 10 years, respectively, relative to expected onset absent intervention (Control Scenario). Under the sustained weight loss scenario (Scenario 1), diabetes onset was 42.2%, 41.1% and 13.7% lower than simulated to occur absent intervention at 3, 5, and 10 years, respectively, following program participation (Table 2). Similar to the NDPP and DPPOS results, the declining percentage reduction in diabetes onset over time (e.g., year 10 versus year 3) reflects that for many people lifestyle changes might delay but not prevent diabetes onset. Among the senior population at risk for CVD, simulated stroke incidence was 15.1% (Scenario 2) to 14.5% (Scenario 1) lower than in the Control Scenario over 5 years (Table 3).

**Table 2:** Outcomes for Population with Prediabetes and Subgroups.

		**Intent-To-Treat**						**Completers** ^a^					
**Observational Outcomes**	**Sample size, n**	997						896					
	**Average weight loss, %**	6.8						7.3					
**Modeled Outcomes**		**Partial weight regain**			**Sustained weight loss**			**Partial weight regain**			**Sustained weight loss**		
		**3-year**	**5-year**	**10-year**	**3-year**	**5-year**	**10-year**	**3-year**	**5-year**	**10-year**	**3-year**	**5-year**	**10-year**
	**Disease onset relative reduction, %**												
	Diabetes	38.1	26.6	11.1	42.2	41.1	13.7	42.2	31.1	8.7	46.1	38.3	17.2
	History of ischemic heart disease	9.3	13.5	11.0	12.2	16.5	13.9	28.6	10.7	13.9	14.5	28.1	16.8
	History of myocardial infarction	15.2	19.6	11.1	22.6	21.3	16.5	27.8	12.2	10.6	19.4	18.5	21.6
	History of congestive heart failure	8.3	14.2	11.6	12.1	15.9	9.6	13.1	12.7	12.5	13.4	15.1	21.1
	History of stroke	14.4	18.2	6.2	18.7	18.1	12.2	14.4	17.6	10.2	18.3	21.4	16.4
	**Per capita medical expenditures saved, $** ^b^	1,950	4,240	12,010	2,100	4,630	14,540	2,400	5,350	14,690	2,550	5,710	16,390
	**Mortality related expenditure relative reduction, %**	7.9	7.6	5.5	8.1	7.7	6.9	7.9	8.2	7.1	6.7	9.0	7.8

Notes:

^a^ Participants completing ≥9 lessons.

^b^ Dollar estimates are present values using a 3% discount rate.

**Table 3:** Outcomes for Population with CVD Risk Factors and Subgroups.

		**Intent-To-Treat**						**Completers** ^a^					
**Observational Outcomes**	**Sample size, n**	1,121						1,003					
	**Average weight loss, %**	6.8						7.3					
**Modeled Outcomes**		**Partial weight regain**			**Sustained weight loss**			**Partial weight regain**			**Sustained weight loss**		
		**3-year**	**5-year**	**10-year**	**3-year**	**5-year**	**10-year**	**3-year**	**5-year**	**10-year**	**3-year**	**5-year**	**10-year**
	**Disease onset relative reduction, %**												
	Diabetes	37.6	28.4	5.3	43.1	35.7	19.5	44.1	32.5	10	44.1	40.8	23.3
	History of ischemic heart disease	9.2	8.7	11.1	11.6	15.9	17.7	13.1	15.5	13.3	10.5	11.3	18.7
	History of myocardial infarction	16.2	19.7	19.8	8.2	17.1	26.5	17.1	21.4	23.1	13.4	23.7	29.1
	History of congestive heart failure	5.1	12.9	13.3	6.6	12.8	20.1	15.1	12.6	16.6	15.5	12.1	17.3
	History of stroke	14.4	15.1	9.2	18.4	14.5	16.2	11.5	23.3	16.1	11.1	19.5	11.1
	**Per capita medical expenditures saved, $** ^b^	1,720	3,840	11,550	1,770	4,240	14,200	1,540	3,710	12,170	1,990	4,710	15,960
	**Mortality related expenditure relative reduction, %**	1.5	3.3	5.8	3.7	47.5	8.2	1.8	4.5	6.1	3.1	6.1	8.4

Notes:

^a^ Participants completing ≥9 lessons.

^b^ Dollar estimates are present values using a 3% discount rate.

Simulation results suggest gross per capita medical expenditures savings of $1,770, $4,240 and $14,200 cumulative over 3, 5 and 10 years, respectively, from the sustained weight loss scenario; savings under the partial weight regain scenario are $1,720, $3,840 and $11,550. Reduced medical expenditures are greater for completers versus all participants (Table 3). In addition, our analysis estimate the 10 year cumulative mortality related expenditures reduce by 6.1% (Scenario 2) to 8.4% (Scenario 1) for completers and 5.8% (Scenario 2) to 8.2% (Scenario 2) for all participants.

**Fig 2:**
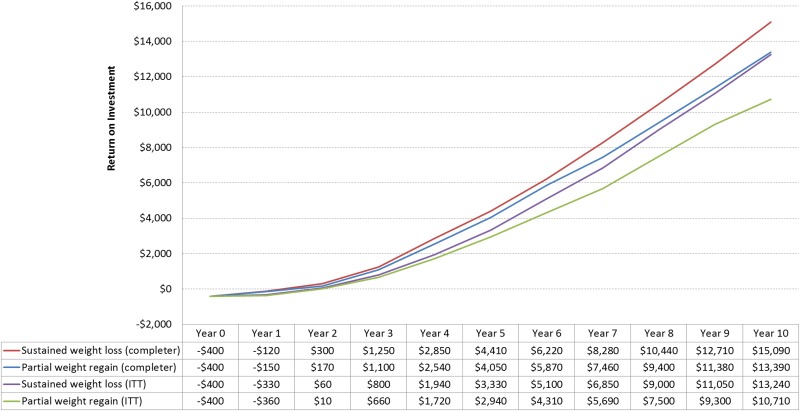
Projected Average Return On Investment of Population with Prediabetes On Digital Intensive Behavioral Counseling Program Participation.

**Fig 3:**
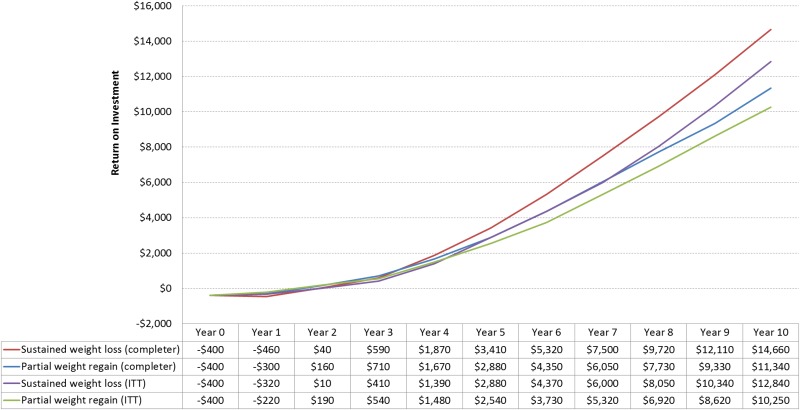
Projected Average Return On Investment of CVD Risk Factor Population On Digital Intensive Behavioral Counseling Program Participation.

For both the population with prediabetes and the entire population with CVD risk factors, medical savings exceed intervention costs after about 1–2 years (Figs 2 and 3). Among all participants, estimated 10-year net savings averaged $10,710 (partial weight regain) to $13,240 (sustained weight loss) for the population with prediabetes, and $10,250 (partial weight regain) to $12,840 (sustained weight loss) for the population at risk for cardiovascular disease.

**Fig 4:**
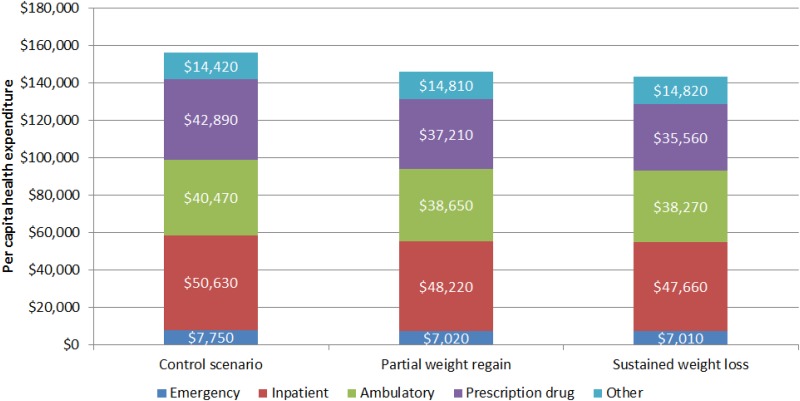
Projected 10-Year Cumulative Health Expenditures, Digital Intensive Behavioral Counseling Program.

The estimated average cumulative medical expenditures over ten years under all three scenarios of seniors with CVD risk factors are shown in Fig 4. On average, 33% of medical spending was for hospital inpatient services, 26% for ambulatory services, 26% for prescription drugs, 5% for emergency care, and 10% for all other costs. Among the estimated 10-year cumulative reduction in medical expenditures, under the partial weight regain scenario, 55% of the savings comes from reduced prescription drug expenditures, 24% from reduced expenditures for hospital inpatient care, 18% from reduced expenditures for ambulatory services and 7% from reduced expenditures for emergency care. Expenditures for the “all other” setting rose by 4%. Similar results were observed under the sustained weight loss scenario (with a slightly higher percentage of savings coming from reduced prescription drug). Likewise, similar results were observed among the senior population with prediabetes.

## Discussion

Previous work has found that IBC intervention, including NDPP, among overweight and obese adults with prediabetes and CVD risk factors can improve clinical measures, prevent or delay disease onset, reduce medical expenditures, and increase productivity (in the form of higher labor force participation and lower work absenteeism) [1–6,13–15,19]. The contribution of this study is that our findings suggest that digital remotely-delivered IBC both effective and cost-saving among a senior population. In particular, this study used weight loss results for senior participants of a digital behavioral counseling program to illustrate potential short term and long term benefits to Medicare beneficiaries.

Our studied senior participants on average reduced body weight by 6.8% over the initial 26 weeks of the program, with program completers averaging 7.3% weight reduction.

Furthermore, 89% of senior participants completed over 9 core lessons compared to only 74% among all adult participants. These results suggest that seniors are able to engage meaningfully with the technology that is an integral part of digital lifestyle interventions that use online access to curricula and support groups, and digital tools such scales and pedometers connected to mobile devices and the Internet.

Furthermore, one of the key outputs of this analysis is the magnitude of diabetes risk reduction. In the model used for this study, assumptions of control-group diabetes incidence are lower than those seen in the NDPP/DPPOS control group. These lower estimates were calculated based on published estimates of diabetes incidence from the CDC, and imply that the relative risk reductions seen in NDPP/DPPOS may be higher than what would be expected in a larger population [1,2]. The net effect of these lower estimates is to render the subsequent cost savings in this study to be more conservative than if the NDPP/DPPOS’s diabetes incidence rates were used.

For seniors with prediabetes, the projected per capita saving over 10 years from achieving the weight reduction results of the digital IBC intervention range from $12,010 to $16,390, depending whether they completed the program and the degree to which they sustained their weight loss. Among the overall senior population with CVD risk factors, cumulative 10-year medical savings ranged from $11,550 to $15,960. Medical savings offset intervention costs at about two years, with cumulative net savings continue to grow over the 10-year projection horizon.

The estimated medical expenditures presented in this study reflect payments from all sources—including patient out-of-pocket expenses and co-insurance payments as well as Medicare payments. Due to the complex structure of Medicare plans and spending under Medicare Part D, the exact proportion of reduced medical expenditures that would accrue to Medicare is unclear. However, on average approximately three-fourths of medical expenditures by Medicare beneficiaries are paid by Medicare [38].

In 2014, 11% of Medicare expenditures were associated with Part D (prescription drugs) [39]. In contrast, the projected prescription drug spending for the overweight and obese Medicare population with prediabetes is 27% of total expenditures. This indicates a strong correlation between weight and drug spending. This correlation is further supported by the finding in our analysis that half of cost savings due to IBC come from the reduction in prescription drug costs. Many chronic conditions associated with obesity are primarily managed by long-term medication treatments, so reducing the prevalence and severity of these chronic diseases reduces the need for medications treating these diseases.

Comparison of these results with published results from other types of weight-loss interventions suggests that digital behavioral counseling has an important role to play in the armamentarium of interventions used to address diabetes and cardiovascular disease incidence via weight loss. For example, Thorpe et al. studied the net savings FDA-approved weight management medications can bring to Medicare through a Dynamic Aging Process (DAP) model [2,3,40]. The authors modeled a scenario with sustained weight loss, as well as a scenario with partial weight regain. For the population with an average BMI ≥27 and at least one additional risk factor (hypertension, type 2 diabetes, and/or dyslipidemia), Thorpe et al. projected that 10% sustained weight loss could generate $8,648 (inflated to 2015 USD) in direct medical savings over 10 years. In our study, for a similar population with BMI ≥24 and CVD risk factors (hypertension, impaired blood glucose, metabolic syndrome, and/or dyslipidemia), the projected savings associated with 6.8% sustained weight loss was $14,200 over 10 years. The higher savings in this study are mainly due to different study cohort, as their sample cohort includes individuals who are overweight with at least one weight-related comorbidity and all obese people who may or may not have other disease condition.

### Study strengths and limitations

In this study we used a previously published and validated microsimulation model to estimate reduced CVD events onset and health expenditure by senior participants of a digital intensive behavioral counseling program. Detailed information on the strengths and limitations of the model are published elsewhere [14,18,19]. Strengths include the use of published data to model the pathways by which improvements in body weight can affect primary (clinical), secondary (disease states), and tertiary (medical expenditures) outcomes. The model allows one to quickly test sensitivity of study findings to the assumption of whether intervention participants might sustain their weight loss.

Study limitations include the following. One, although the senior participants in the digital IBC program may represent those who chose to voluntarily participate in a digital intervention program, they may not necessarily reflect the characteristics of the general Medicare beneficiaries. However, as Medicare beneficiary participation in a program like this would be voluntary, it is plausible to expect similar benefits among participants in a broader Medicare population for whom the benefit is covered. Two, weight loss was the only clinical data collected from the digital IBC participants; all other starting clinical values required to run the simulation were created by using propensity matching (based on age, sex, race/ethnicity, BMI, and presence of prediabetes or other CVD risk factors) with NHANES records. Three, the medical expenditure equations used in the model reflect total expenditures (including Medicare payments, patient out-of-pocket expenses, and payments from supplementary insurance). Four, limitations of the model include use of some older data sources and non-US data sources (though extensive model validation and calibration was completed to ensure that simulation results were consistent with published outcomes).

## Conclusion

This study shows that a digital, remotely-delivered intensive behavioral counseling (IBC) program can help seniors who display risk factors for diabetes and cardiovascular disease achieve significant weight loss. Modeled impacts of these study results suggest that this digital IBC program reduces the risk for diabetes and cardiovascular disease, leading to net savings of $10,250 to $12,840 over 10 years. These findings affirm recommendations for coverage of IBC by the U.S. Preventive Services Task Force [3].

## Supporting Information

S1 Table Data Sources for Key Model Biometric Parameters. (DOCX)

S2 Table Regression Results for Estimating Total Annual Medical Expenditures. (DOCX)

S3 Table Regression Results for Allocation of Medical Expenditures. (DOCX)
