# Supplementary material for: Clinical and Economic Impact of a Digital, Remotely-Delivered Intensive Behavioral Counseling Program on Medicare Beneficiaries at Risk for Diabetes and Cardiovascular Disease
Source: PLoS One. 2016 Oct 5;11(10):e0163627. doi: 10.1371/journal.pone.0163627 (PMC5051965; doi:10.1371/journal.pone.0163627)
Supplement: S1 Table — (DOCX) [file pone.0163627.s001.docx]

**S1 Table. Data Sources for Key Model Biometric Parameters**

| **Outcome** | **Outcome variable** | **Correlation (equation)** | **Source** |
| --- | --- | --- | --- |
| **Systolic Blood Pressure** | Annual SBP Change due to change in BMI | 1kg loss in body weight was associated with a 1.05 mmHg reduction in SBP | Neter et al., 2003 [1] |
|  | Annual SBP Change due to aging (female) | 107.16+0.5027×age + 0.0034×age^2^ | Analysis of 2003-2010 NHANES |
|  | Annual SBP Change due to aging (male) | 118.11+0.2147×age +0.0032×age^2^ |  |
| **Diastolic Blood Pressure** | Annual DBP Change due to change in BMI | 1kg loss in body weight was associated with a 0.92 mmHg reduction in DBP | Neter et al., 2003 [1] |
|  | Annual DBP Change due to aging (female) | 0.5881+0.0177×age - 0.0025×age^2^+ 0.00003×age^3^ | Analysis of 2003-2010 NHANES |
|  | Annual DBP Change due to aging (male) | 0.3808+ 0.0268×age - 0.0021×age^2^+0.00002×age^3^ |  |
| **HbA1C Level** | Annual change in HbA1C level | an average change of 1kg in weight was associated with a 0.071% change in HbA1c levels | Heianza et al., 2012a [2]  Heianza et al., 2012b [3] |
| **HDL cholesterol** | Annual HDL change due to aging (male) | -0.1722+(0.0354×(age-20))+(-.0048×((age-20) ^2^))+(.0002×((age-20) ^3^))+(-.000004×((age-20) ^4^))+(.00000003×((age-20) ^5^)) | Framingham Heart Study, Wilson et al., 1994 [4]  Analysis of 2003-2010 NHANES |
|  | Annual HDL change due to aging (female) | -(0.2674) + (0.0027×(age-20)) - (0.0006×((age-20)×(age-20)))+(.000007×((age-20) ^3^)) |  |
|  | Annual HDL change due to change in BMI (male) | -0.0724 - (0.0007×(age-20)) +(0.00001×((age-20)×(age-20))) |  |
|  | Annual HDL change due to change in BMI (female) | -(0.1224) + (0.0044×(age-20)) -(0.00001×((age-20)×(age-20))) |  |
| **Total cholesterol** | Annual T-C change due to aging (male) | 1.6486 - (0.0587×(age-20)) + (0.00008×((age-20)×(age-20))) + (0.000003×((age-20) ^3^)) | Framingham Heart Study, Wilson et al., 1994 [4]  Analysis of 2003-2010 NHANES |
|  | Annual T-C change due to aging (female) | 0.144+(0.2043×(age-20))-(0.0068×((age-20)×(age-20)))+(0.00005×((age-20) ^3^)) |  |
|  | Annual T-C change due to change in BMI (male) | 0.6624 - (0.004×(age-20)) - (0.00006×((age-20)×(age-20))) |  |
|  | Annual T-C change due to change in BMI (female) | 0.2942 - (0.0098×(age-20)) + (0.0003×((age-20)×(age-20))) |  |

Abbreviations: NHANES, the National Health and Nutrition Examination Survey.

Reference

1. Neter JE, Stam BE, Kok FJ, Grobbee DE, Geleijnse JM (2003) Influence of weight reduction on blood pressure a meta-analysis of randomized controlled trials. Hypertension 42: 878-884.
2. Heianza Y, Arase Y, Fujihara K, Tsuji H, Saito K, Hsieh SD, Kodama S, Shimano H, Yamada N, Hara S (2012) Screening for pre-diabetes to predict future diabetes using various cut-off points for HbA1c and impaired fasting glucose: the Toranomon Hospital Health Management Center Study 4 (TOPICS 4). Diabetic Medicine 29: e279-e285.
3. Heianza Y, Arase Y, Fujihara K, Hsieh SD, Saito K, Tsuji H, Kodama S, Yahagi N, Shimano H, Yamada N (2012) Longitudinal Trajectories of HbA1c and Fasting Plasma Glucose Levels During the Development of Type 2 Diabetes The Toranomon Hospital Health Management Center Study 7 (TOPICS 7). Diabetes care 35: 1050-1052.
4. Wilson PW, Anderson KM, Harri T, Kannel WB, Castelli WP (1994) Determinants of change in total cholesterol and HDL-C with age: the Framingham Study. Journal of gerontology 49: M252-M257.
