# Supplementary material for: Clinical and Economic Impact of a Digital, Remotely-Delivered Intensive Behavioral Counseling Program on Medicare Beneficiaries at Risk for Diabetes and Cardiovascular Disease
Source: PLoS One. 2016 Oct 5;11(10):e0163627. doi: 10.1371/journal.pone.0163627 (PMC5051965; doi:10.1371/journal.pone.0163627)
Supplement: S2 Table — (DOCX) [file pone.0163627.s002.docx]

**S2 Table. Regression Results for Estimating Total Annual Medical Expenditures**

|  | **Age less than 65** | | | | **Age 65 and over** | | | |
| --- | --- | --- | --- | --- | --- | --- | --- | --- |
|  | **Non obese** | | **Obese** | | **Non obese** | | **Obese** | |
| **Parameter** | **GLM Coefficient** | **Pr > ChiSq** | **GLM Coefficient** | **Pr > ChiSq** | **GLM Coefficient** | **Pr > ChiSq** | **GLM Coefficient** | **Pr > ChiSq** |
| **Intercept** | 6.2076 | <.0001 | 5.9581 | <.0001 | 7.4378 | <.0001 | 6.749 | <.0001 |
| **Male** | -0.2912 | <.0001 | -0.4563 | <.0001 | -0.0748 | 0.0014 | -0.2124 | <.0001 |
| **Age** |  |  |  |  |  |  |  |  |
| **35 to 44** | 0.2707 | <.0001 | 0.1502 | <.0001 |  |  |  |  |
| **45 to 64** | 0.4911 | <.0001 | 0.4141 | <.0001 |  |  |  |  |
| **65 to 74** |  |  |  |  | -0.2208 | <.0001 | -0.0149 | 0.6662 |
| **Race/ethnicity** |  |  |  |  |  |  |  |  |
| **Black** | -0.4475 | <.0001 | -0.4456 | <.0001 | -0.0806 | 0.1235 | -0.0681 | 0.2933 |
| **Other race** | -0.3503 | <.0001 | -0.3629 | <.0001 | -0.0488 | 0.4001 | 0.1718 | 0.1441 |
| **Hispanic** | -0.4121 | <.0001 | -0.3543 | <.0001 | -0.2202 | <.0001 | -0.1235 | 0.0256 |
| **Insured** | 0.6703 | <.0001 | 0.7603 | <.0001 | 1.4213 | <.0001 | 1.6855 | <.0001 |
| **Insured through Medicaid** | -0.1544 | <.0001 | 0.3201 | <.0001 |  |  |  |  |
| **Body weight** |  |  |  |  |  |  |  |  |
| **Overweight** | -0.0169 | <.0001 |  |  | -0.086 | 0.0002 |  |  |
| **Continuous BMI (for Obese adults)** |  |  | 0.0118 | <.0001 |  |  | 0.0158 | <.0001 |
| **Disease presence** |  |  |  |  |  |  |  |  |
| **Hypertension** | 0.3437 | <.0001 | 0.4009 | <.0001 | 0.2468 | <.0001 | 0.0414 | 0.3281 |
| **Diabetes** | 0.8477 | <.0001 | 0.7068 | <.0001 | 0.3887 | <.0001 | 0.353 | <.0001 |
| **Congestive heart failure** | 1.8533 | <.0001 | 1.2012 | <.0001 | 1.0504 | <.0001 | 0.7898 | <.0001 |
| **Ischemic heart disease** | 1.1771 | <.0001 | 1.1101 | <.0001 | 0.6487 | <.0001 | 0.5207 | <.0001 |
| **History of stroke** | 0.8110 | <.0001 | 0.3661 | <.0001 | 0.4246 | <.0001 | 0.3629 | <.0001 |
| **History of myocardial infarction** | 1.1858 | <.0001 | 1.1731 | <.0001 | 0.7013 | <.0001 | 0.6061 | <.0001 |
| **Retinopathy** | 0.7041 | <.0001 | 0.6623 | 0.0006 | 0.6043 | <.0001 | 0.2479 | 0.1458 |
| **Renal failure** | 2.7283 | <.0001 | 2.1572 | <.0001 | 1.4409 | <.0001 | 1.6285 | <.0001 |
| **Interactions with diabetes** |  |  |  |  |  |  |  |  |
| **Congestive heart failure** | -0.5183 | 0.1842 | -0.3229 | 0.2001 | -0.1852 | 0.3232 | 0.1807 | 0.7166 |
| **Ischemic heart disease** | -0.3496 | 0.0361 | -0.1973 | 0.1109 | -0.1444 | 0.0515 | 0.0853 | 0.5399 |
| **History of myocardial infarction** | -0.2685 | 0.2147 | -0.3629 | 0.0261 | -0.1292 | 0.1874 | 0.1111 | 0.7992 |
| **History of stroke** | -0.1942 | 0.2303 | 0.0910 | 0.5154 | 0.1341 | 0.0863 | 0.0912 | 0.1816 |
| **Renal failure** | -1.0415 | 0.0107 | -0.8523 | 0.04 | 0.4487 | 0.142 | 0.3442 | 0.2593 |
| **Fit statistics:** |  | | | | | | | |
| **N** | 93,411 | | 24,826 | | 10,570 | | 4,374 | |
| **Deviance/DF:** | 43,894.00 | | 37,316 | | 17,264 | | 13,363 | |
| **Scaled deviance/DF:** | 1.297 | | 1.285 | | 1.26 | | 1.23 | |
| **Pearson Chi-square/DF:** | 3,343,301 | | 510,174 | | 34,431 | | 18,761 | |
| **Scaled Pearson Chi-square/DF:** | 15.4 | | 17.6 | | 2.5 | | 1.7 | |

Note: Results of GLM model with gamma distribution and log link.
