# Supplementary material for: Clinical and Economic Impact of a Digital, Remotely-Delivered Intensive Behavioral Counseling Program on Medicare Beneficiaries at Risk for Diabetes and Cardiovascular Disease
Source: PLoS One. 2016 Oct 5;11(10):e0163627. doi: 10.1371/journal.pone.0163627 (PMC5051965; doi:10.1371/journal.pone.0163627)
Supplement: S3 Table — (DOCX) [file pone.0163627.s003.docx]

**S3 Table. Regression Results for Allocation of Medical Expenditures**

|  | **Model parameter estimate** | | | | | | | |
| --- | --- | --- | --- | --- | --- | --- | --- | --- |
|  | **Emergency** | | **Inpatient** | | **Ambulatory** | | **Prescription drug** | |
| **Coefficients** | **Log-ratio** | **Logistic** | **log-ratio** | **Logistic** | **Log-ratio** | **Logistic** | **Log-ratio** | **Logistic** |
| **Intercept** | 1.6257** | -2.7869** | 4.0254** | -3.0416** | 1.9275** | 0.1947** | 1.2800** | -0.0984* |
| **Age 18 to 34** | 1.3526** | 0.1956** | 1.6755** | -0.1508** | -0.0719 | -0.7639** | -0.8794** | -0.9023** |
| **Age 35 to 44** | 1.5597** | -0.0148** | 1.1773** | -0.5261** | -0.1038 | -0.6117** | -0.6817** | -0.7868** |
| **Age 45 to 64** | 1.1497** | -0.3034** | 0.8203** | -0.7007** | -0.4016** | -0.4485** | -0.7479** | -0.6136** |
| **Age 65 to 74** | 0.3993 | -0.2114 | 0.2763 | -0.2041** | 0.0015 | -0.2038** | -0.0627 | -0.2011** |
| **Hispanic** | 0.5988* | 0.2485** | 0.5374 | 0.1646** | 0.7314** | -0.1382** | 0.5004** | -0.0321 |
| **Black** | 0.5629* | 0.5302** | 0.4120 | 0.2757** | 0.4856** | -0.2011** | 0.3389** | 0.0070 |
| **White** | -0.3465 | 0.4275** | -0.5426 | 0.3487** | -0.0639 | 0.5090** | -0.0607 | 0.6714** |
| **Normal weight** | -0.4712** | -0.0373 | -0.1855 | -0.0011 | -0.2907** | -0.0788** | -0.2543** | -0.1248** |
| **Obese** | 0.1289 | 0.1420** | -0.0238 | 0.0669* | 0.1379* | 0.0651** | 0.1919** | 0.1345** |
| **Male** | 0.4178** | -0.2985** | 0.2779 | -0.6011** | 0.0278 | -0.8146** | 0.1546** | -0.7661** |
| **Smoker** | 0.9508** | 0.4164** | 0.9071** | 0.0410 | 0.8251** | -0.2010** | 0.8521** | 0.0679** |
| **Presence or history of** |  |  |  |  |  |  |  |  |
| **Hypertension** | -0.1248 | 0.3125** | -0.2492 | 0.3404** | 0.4362** | 0.7276** | 0.8444** | 1.2579** |
| **Cardiovascular disease** | -0.5035** | 0.5142** | 0.1009 | 0.6220** | 0.0553 | 0.5937** | 0.0622 | 0.7397** |
| **Heart attack** | 0.1725 | 0.1941** | -0.2486 | 0.5041** | 0.3384** | 0.0653 | 0.6114** | 0.2733** |
| **Stroke** | -0.7694** | 0.5802** | -1.0009** | 0.6067** | -0.3254** | 0.2007** | -0.1359 | 0.5735** |
| **Diabetes** | -0.1345 | 0.2442** | -0.0440 | 0.3774** | 0.3522** | 0.8885** | 1.1373 | 1.7220** |
| **Arthritis** | -0.6162** | 0.3974** | -0.7568** | 0.3753** | 0.1743** | 0.8168** | 0.1664** | 0.9026** |
| **Insured** | -1.0678** | 0.0162 | -0.9866** | 0.2749** | -1.2608** | 1.2191** | -1.3034** | 0.8902** |
| **Has Medicaid** | -0.2507 | 0.7074** | 0.2682 | 0.8279** | 1.0365** | 0.1247** | 1.4200** | 0.2885** |
| **Fit statistics (N=121,360)** |  | | | | | | | |
| **-2 Log Likelihood** | 186,711 | | 122,139 | | 654,374 | | 620,176 | |
| **Akaike information criterion (AIC)** | 186,793 | | 122,221 | | 654,456 | | 620,258 | |
| **Bayesian information criterion (BIC)** | 187,191 | | 122,619 | | 654,854 | | 620,656 | |
| **Pearson Statistic** | 120,099 | | 115,370 | | 120,557 | | 123,202 | |

Note: ** Statistically significant at the 0.01 level. * Statistically significant at the 0.05 level Omitted categories include age 75 and above, “other” race/ethnicity and overweight. All variables in the model are dichotomous (1=characteristic present, 0=characteristic not present).

The zero inflated log-ratio regression approach used data on all adults in MEPS with the same explanatory variables used to model total annual medical expenditures. Separate regressions were estimated for ambulatory, inpatient, emergency, and prescription drug categories (Table 2). The dependent variable for each regression reflected the log of the ratio of category expenditures to expenditures in the “all other” category (e.g. log of ratio of inpatient expenditures to all other expenditures). There are two components from the regression outcome. One component involved estimating logistic regressions to model the probability an individual incurred expenditures in that category. The second component analyzed the log transformed ratio using general linear model with the same explanatory variables. Combining the information from 2 components allowed us to calculate the proportion of each person’s total annual medical expenditures across the five cost categories
